# Supplementary material for: Are carbon emissions trading and green financial instruments synergistic? -Comprehensive quantitative research based on content analysis
Source: PLoS One. 2024 Mar 7;19(3):e0298601. doi: 10.1371/journal.pone.0298601 (PMC10919601; doi:10.1371/journal.pone.0298601)
Supplement: S1 Data — (ZIP) [file pone.0298601.s001.zip › Data/地级市Z_F工作报告.zip!/┴╔─■/╔≥╤⌠╩╨/└┤╫╘╣1⁄2╓┌║┼í╛┬φ┐╦╩2╛▌═°í┐_2016.docx]

各位代表：

　　现在，我代表市政府向大会报告工作，请予审议，并请市政协各位委员提出意见。

　　一、2015年和“十二五”时期主要工作回顾

　　过去一年，面对艰巨繁重的改革发展任务和经济下行压力，在市委的坚强领导下，在市人大及其常委会依法监督和市政协民主监督下，市政府紧紧依靠全市人民，认真贯彻落实党的十八大及十八届三中、四中、五中全会和习近平总书记系列重要讲话精神，科学把握发展趋势，坚持稳中求进工作总基调，主动适应引领经济发展新常态，拼搏进取，攻坚克难，推动全市经济社会实现了平稳健康发展。

　　预计全市地区生产总值实现7280亿元，比上年增长3.5%；固定资产投资完成5350亿元，下降18.5%；一般公共预算收入完成606.2亿元，下降22.8%；社会消费品零售总额实现3885亿元，增长8.2%；城镇居民人均可支配收入达到36664元，增长7.1%；农村居民人均可支配收入达到13498元，增长7.8%；城镇登记失业率为3.18%。

　　(一)经济保持稳定增长

　　坚持把稳增长作为经济工作重中之重，综合施策、主动作为，经济发展动力逐步转换，政策效力进一步显现。

　　大力培育发展动力。深入实施创新驱动发展战略，成立中科院智能制造及机器人技术创新与产业化联盟，组建机器人协同创新中心，国家机器人检测与评定中心、质量监督检验中心获批并建设，新增2个国家级重点实验室。中科仪泵阀项目通过国家重大科技专项验收，沈鼓集团研发出国内首台10万立方米空分压缩机组，国内首款锐翔电动轻型飞机批量生产，机床集团i5系列智能机床销量增长1.5倍，沈阳高新区成为国家首批科技服务业试点，全市规模以上工业高新技术产品增加值实现865亿元，科技创新对经济发展支撑作用进一步增强。积极推进产业创新，大力培育新产业、新模式、新优势，出台《沈阳市优先培育新兴业态指导意见》，加大专业部门抓产业力度，战略性新兴产业加快布局，新型服务业态蓬勃发展。

　　充分挖掘增长潜力。注重发挥有效投资关键作用，狠抓重大项目建设，全市开复工投资亿元以上项目1401个、完成投资3603.2亿元，270个市级重点项目完成投资1643.2亿元，255个政府投资项目完成投资124.2亿元。积极开展政府和社会资本合作，向社会公布89个PPP项目，总投资1653亿元，65个重点项目实现对接，华夏幸福公司投资64亿元整体开发建设沈水生态创新科技城，大辛垃圾焚烧发电等5个项目成为国家示范项目，非公投资占固定资产投资比重为84%。注重发挥消费基础性作用，制定促进旅游、会展、养老、文化、住房等消费政策措施，积极培育新增长点，消费对经济增长贡献率进一步提高。注重发挥出口促进作用，积极建设外贸出口通道和平台，成立“走出去”企业合作联盟，实施东北区域通关一体化，全市出口总额70亿美元。经济要素指标平稳增长，全社会用电量增长3.7%，货运总量增长5.9%；居民消费价格上涨1.2%。

　　积极发挥政策效力。深入贯彻落实国家新一轮东北振兴战略和稳增长各项政策措施，“三争取”工作成效显著，争取到国家和省重大项目153个、政策46项、专项资金总额439.5亿元，中德装备园成为全国首个由国务院批准的装备制造业对德合作园区。深入开展“双进双解”活动，帮扶企业1765家、重点项目320个。停止收取建设工程的安措费、社保费等，减轻企业负担26.6亿元。出台“大众创业、万众创新”实施意见和75条政策措施，与瀚华金控合作设立中小微企业融资担保基金和债权基金。推进商事制度改革，全市新登记公司2.6万户，增长13.5%，我市成为国家小微企业创业创新基地示范城市。

　　(二)转型升级取得明显成效

　　坚持把提高质量效益放在突出位置，着力促进产业结构调整，三次产业结构进一步优化。

　　工业结构加快调整。积极推动传统产业转型升级，制定《沈阳“中国制造2025”实施方案》，实施制造业智能升级三年行动计划，组织千名企业家进行智能制造专题培训，100户规模以上企业开展智能改造升级，首批20户示范企业已完成咨询诊断和解决方案编制，机床集团、东软集团改造项目成为国家智能制造试点示范项目，沈鼓集团、特变电工等骨干企业高端产品产值占60%以上。实施优势企业倍增计划，制定我市优质产品和促进企业间相互配套支持政策，汽车及零部件、机械装备等优势产业加快发展，规模以上装备制造业增加值占工业比重提高2.4个百分点。淘汰压缩了落后过剩产能。大力发展新兴产业，智能制造、航空装备、生物制药等产业规模不断壮大，50个重点培育项目加快推进。东软医疗、诺康生物、芯源微电子等企业产值增长10%以上。以新松为龙头的机器人产业迅猛发展，业务收入实现55亿元，增长30%。

　　服务业提质增效。深入实施服务业发展四年行动计划，服务业增加值占比提高2个百分点，现代服务业比重达到54.2%。渣打银行、友利银行等20家区域金融总部在沈设立。三生制药、桃李面包等18家企业上市、挂牌，新增贷款突破1300亿元，金融业增加值增长22.8%。出台促进会展业发展实施方案，成功举办中国绿公司年会、制博会暨机器人展览会、东北文博会、智慧产业博览会、法库国际飞行大会等大型会展活动，会展交易额增长7.9%。工业企业分立生产性服务企业170户。积极促进旅游业改革发展，沈阳成为境外旅客购物离境退税城市，全年接待游客增长11%，旅游总收入增长14.6%。消费市场繁荣活跃，商贸物流、电子商务、电信服务、快递业发展迅速，限额以上企业网上零售额增长50%。我市成为国家综合运输服务示范市，和平区成为国家电子商务示范区，永安新城电子商务产业园成为国家级示范基地。出台房地产扶持政策，商品房销售1640.5万平方米，增长13.1%，其中外地人购房占45.6%，二手房销售面积增长44.7%，房地产业实现平稳健康发展。

　　现代农业加快发展。充分发挥龙头企业带动作用，积极调整农业结构，大力发展多种形式适度规模经营，全市土地流转面积达到266.8万亩，新增农业产业化龙头企业17家。辽中县成为国家地理标志产品保护示范区，法库县成为国家农产品质量安全示范县。落实各项强农惠农政策，持续加强农业基础设施建设，粮食生产实现“十二连丰”。蒲河生态经济带93个市级重点项目完成投资1494亿元，沈康现代农业示范带32个农业科技园和10个休闲农业园区加快建设，环城都市农业产业带建成38个休闲采摘园、形成4条精品旅游线路，“三带”建设有力促进了县域经济发展。

　　(三)改革开放扎实推进

　　坚持以改革开放促进经济社会发展，突出问题导向，下大气力破除体制机制障碍，经济社会发展活力进一步增强。

　　重点领域改革取得新突破。通过积极努力，沈阳成为全国8个全面创新改革试验区之一。深入推进简政放权，累计取消、下放行政职权1089项，政府权责清单向社会公布，精简行政职权61.7%。进一步优化审批流程，减少审批要件2047个，削减31.5%；将基本建设审批环节由31个减少到17个，压缩45.2%。加快推进国企改革，机床集团、副食集团成功与社会资本组建混合所有制企业，北方重工与中建材实现战略重组。统筹城乡改革取得新成果，于洪区成为国家中小城市综合改革试点地区，新民市获批国家新型城镇化综合试点市，康平县被列为国家基础设施投融资体制改革试点县。优化金融生态改革试验深入推进，中兴通讯金融研究院在沈设立，东北首家农村综合产权交易中心挂牌运营，全市金融及服务机构达745家，金融市场活力进一步增强。投融资及财税体制改革稳步推进，设立规模100亿元的振兴发展基金，财政资金杠杆和引导作用得到充分发挥。

　　对外开放迈上新台阶。加大对境内外重点产业招商引资力度，全年引进内资867亿元，利用外资11亿美元。中德装备园与德国工业4.0联盟全面合作，西门子、诗道芬等德国企业积极参与我市产业发展、制造业转型、基础设施建设，对德合作迈入新阶段。深入实施“走出去”战略，积极开展国际产能合作，我市企业在印度、乌干达、白俄罗斯等国家建立项目基地，北方重工、联立铜业分别在境外拿到6亿美元和8亿美元项目大单，“沈满欧”班列开辟了对外经贸合作新通道，全市新签对外投资协议额、承包工程合同额分别为5.7亿美元和17.57亿美元。成功举办德国企业沈阳行暨中德智能制造创新论坛、“韩国周”等大型经贸活动，城市知名度和影响力大幅提升。

　　区域协同发展实现新提升。加强与沈阳经济区各城市联动发展，在基础设施、社会事业、产业发展、共同市场等方面对接合作，成立沈阳经济区旅游企业联盟、文化产业一体化联盟和城市商业银行联盟，“乘车易”在沈阳经济区范围内启用，沈抚同城化取得新进展。

　　(四)智慧城市建设全面展开

　　遵循惠民、兴业、善政原则，大力推进信息化与城市化融合发展，智慧城市建设取得初步成效，城市现代化治理能力不断提高。

　　智慧城市建设总体布局基本完成。编制完成沈阳市智慧城市总体规划、智慧产业发展规划和智慧沈阳建设实施方案。组建大数据管理局和运营公司，创建东北大数据产业联盟、智慧城市研究院、大数据研究院和大数据应用创新中心，大数据创新展示馆正式揭牌，设立大数据产业投资基金，管理体系、政策体系、标准体系初步形成。

　　智慧城市基础设施逐步完善。注重发挥东网超云平台作用，依托移动、联通、电信三大运营商加快宽带城市、光网城市建设，主城区光纤网络覆盖率达到99.3%，4G无线网络覆盖率达到99%，智能WiFi网络热点覆盖按计划推进，大数据采集、加工、利用、共享统一平台启动建设。

　　智慧城市重点工程加快实施。启动智慧政务、智慧医疗、智慧教育、智慧交通、智慧社区等11项重点工程。依托96123和“沈阳新社区”等网络服务平台，构建了三级市民智能服务网络。“我的沈阳”智能门户和“行车易”、“停车易”、“乘车易”、“就业通”等上线运行，中街智慧商圈、西塔智慧街和东中街智慧云街投入应用，首家“云医院”启动运营，300所中小学建成数字校园。加速培育智慧产业，阿里、百度、中兴、浪潮、软通动力等互联网龙头企业项目纷纷落地。全市统一协同办公平台和网上并联审批平台正式运行，行政效能和服务水平明显提高。

　　(五)城市建设管理水平进一步提升

　　更加注重城市功能完善和城乡环境治理，全年完成城建投资300亿元，城乡面貌明显改观。

　　畅通工程加快建设。地铁4号线、南北快速干道、五爱立交桥开工建设，地铁9号、10号和2号线北延长线工程进展顺利，二环快速路(二期)、迎宾路高架桥、东陵路高架桥建成通车，沈阳北站北广场、沈阳南站等一批城市重点交通工程、基础设施项目建成并投入使用。加快创建“公交都市”，新建停车场(站)16处，增加新能源公交车500辆，新开调整公交线路38条，公交出行率提高到43%。加快城际交通发展，京沈客专建设积极推进，沈丹高铁、沈康高速三期建成通车，城市交通体系不断完善。

　　蓝天工程扎实推进。开展9家火电企业和240台套燃煤锅炉脱硫、脱硝、除尘改造，120台燃煤锅炉实现清洁能源替代，拆除供热锅炉270台。综合整治扬尘污染，城区主干道机械化作业比重、工地围档设置比率大幅提高。加强机动车尾气治理，大力推广使用新能源汽车，淘汰黄标车3.4万台，新增加气站25座。加强秸秆焚烧治理和管控，秸秆综合利用率提高5%。加快污染企业关停和搬迁改造，节能减排各项目标全面完成。

　　绿化提升行动稳步实施。制定实施绿化提升三年行动计划，按照“身边有绿、出门见绿”目标，开展城市出口路、重要街路、公园景观提升及小区绿化工程，完成舍利塔碑林公园和万泉、仙女湖等10处公园改造，实施5条出口路景观提升、50块节点绿地绿化、389条街路行道树补植，新增17条绿篱街路、8条特色花街，全市植树造林26.1万亩，城乡绿化水平不断提高。

　　城乡环境持续改善。以巩固“创城”成果为主线，扎实推进市容环境综合整治。市区共投入1.5亿元，新增环卫设备214台，大力实行“绿色除雪”。美丽宜居乡村建设取得阶段性成果，投资12亿元完成5个示范乡镇、61个示范村建设，全年新建、改造、维修农村公路688公里。卧龙湖生态保护利用取得重要成果，康平辽河、辽中蒲河两个国家湿地公园通过验收。老虎冲垃圾焚烧发电项目开工建设、渗沥液处理厂建成运行。完成131.4公里供水、供热管网改造，国家地下综合管廊试点全面启动。

　　(六)社会更加和谐稳定

　　以百姓需求为导向，全力推进各项民生工程，重点民生支出比重达到85%，发展成果更多更好地惠及了人民群众。

　　就业和社会保障工作成效显著。启动“万人创业工程”，设立5000万元大学生及青年创业就业发展基金，开展“双创”系列活动，实名制就业16.5万人，新增创业人员1.3万人。完善社会保险和社会救助制度，企业退休人员基本养老金月人均提高209.7元，城乡居民基础养老金提高到月均145元，新农合补助标准提高到人均380元，城乡低保标准分别提高7.4%和12.7%。开展“城乡互联、结对共建”，推进精准扶贫脱贫，贫困人口减少3.6万人。完成保障性住房1.4万套(户)、棚户区改造4.1万套(户)、农村危房改造659户，改建农村厕所2.6万座。830个旧住宅区环境提升、1071万平方米节能暖房改造、20.74万农民饮水安全工程建设等民生实事全部完成。

　　社会事业全面发展。教育强区县创建工作全面完成，在省内率先通过全域义务教育发展均衡县国家验收，新增1万个普惠性幼儿园学位，完成230所中小学标准化建设，新建72所农村中小学塑胶操场，我市成为国家教育管办评分离改革试点市。推进文化惠民工程，开展“百万市民”走进博物馆系列活动，规划建设“抗战历史”文化遗产保护工程，东北陆军讲武堂旧址陈列馆、皇姑屯事件历史博物馆建成开放，辽文化博物馆投入使用，八一公园改造成为国防教育主题公园，公共文化服务体系逐步完善。全面推进医改工作，提前一年在全省率先完成县级公立医院改革。整合优质医疗资源，组建9个“医联体”，推行多点执业和分级诊疗，国家基层中医药先进市通过检查验收。启动“医养结合”新型养老服务模式试点，10个区域性居家养老服务中心和77个农村幸福院投入使用。慈善和残疾人工作扎实推进，民族宗教事业和谐健康发展。“足球之都”建设成效明显，建立足球产业孵化基地，改造建设137块足球场，129个校园足球场地向社会开放，法库获批全国青少年校园足球试点县。我市成为国家体育产业试点城市，全民健身“十百千万”工程蓬勃开展，“沈阳马拉松”成功举办，营造了注重健康、热爱体育、崇尚运动的浓厚氛围。

　　社会治理水平不断提高。实施和谐社区建设工程，市区两级财政投入2.88亿元，完成282个城乡社区公共用房达标改造等项目，启动建设3个区域性社区服务中心。积极开展信访积案攻坚行动，社会矛盾妥善化解。严厉打击违法犯罪行为，安全生产、质量监督、食药监管、交通消防、防灾减灾、应急处置工作进一步加强，我市通过首个国家级安全城区现场评定，“平安沈阳”建设取得明显成效。高标准推进双拥模范城创建。经过不懈努力，我市成为全国文明城市。

　　(七)政府自身建设取得新进展

　　开展行政效能提升行动，不断增强政府系统学习力、执行力、创新力，政府服务能力和水平逐步提高。

　　学习型政府建设不断深入。加强政府各级干部学习和培训，围绕智慧城市、产业转型、创新发展、法治政府建设等重点工作，聘请专家学者专题授课，组织政府干部赴先进地区调研学习。开展市政府智库建设，政府决策的科学化、民主化水平不断提高。

　　法治政府建设不断加强。自觉接受市人大及其常委会的依法监督、市政协的民主监督和社会各方面监督，人大代表建议、政协提案全部办复，满意率不断提高。出台《深入推进依法行政加快建设法治政府意见》，建立重大决策合法性审查制度，进一步规范行政自由裁量权和行政执法检查权。加强政府法制机构和队伍建设，政府依法决策、依法行政水平进一步提高。

　　廉洁政府建设扎实推进。认真践行“三严三实”和“讲诚信、懂规矩、守纪律”要求，制定出台《市政府党组关于全面落实党建工作和党风廉政建设责任制规定》，全面落实主体责任、监督责任，寓审计监督于服务保障发展之中，惩治和预防腐败体系进一步健全，廉洁政府建设走向制度化。

　　效能政府建设取得实效。继续开展“转职能、转作风、优环境”专项行动，坚持“三个导向”、推行“三化”方法，引导干部树立“七种精神”，积极开展“不作为、不担当”问题专项整治，加强政策执行等重要事项督查督办，政府行政效能进一步提升。

　　各位代表，刚刚过去的2015年，是“十二五”收官之年。五年来，全市上下积极应对挑战，抢抓发展机遇，齐心协力，锐意进取，励精图治，砥砺前行，推动沈阳老工业基地振兴发展取得了阶段性重要成果。

　　经济持续稳定发展，产业结构进一步优化。地区生产总值突破7千亿元，年均增长8.1%；一般公共预算收入累计完成3527.9亿元，年均增长5.4%。高新技术产业、新兴产业和现代服务业对经济增长贡献进一步加大，服务业增加值占比提高2.6个百分点。

　　改革开放不断深化，发展动能进一步增强。扎实推进新型工业化综合配套改革，大力实施创新驱动和对外开放战略，经济社会发展动力和活力进一步增强。世界500强企业在沈投资项目112个，五年实际利用外资226.8亿美元，实际到位内资、进出口总额分别突破5000亿元和600亿美元。

　　城市功能持续完善，承载能力进一步提升。编制完成新一轮沈阳城市总体规划和土地利用规划，优化城市空间布局，实施行政中心南迁，城市功能分区更加合理。以承办“十二运”为契机，持续加大城市建设和管理力度，累计完成城建投资2000亿元，城市功能进一步完善、配套更加齐全，区域中心城市集聚辐射能力显著增强。

　　宜居之都建设取得实效，生态环境进一步改善。强化辽河、蒲河、棋盘山、卧龙湖生态保护治理，重点项目全面完成。全市城区绿化覆盖率达到42.2%，城市污水处理率达到95%，生活垃圾处理率达到100%，“十二五”节能减排目标任务超额完成。

　　社会建设稳步推进，人民生活水平进一步提高。每年坚持为城乡居民办一批实事，财政每年民生投资占公共预算支出比重稳步提高。城乡居民收入分别是2010年的1.65倍和1.72倍，社会保障体系逐步完善，百姓幸福感不断增强。

　　各位代表，过去五年，是成绩显著的五年，是硕果累累的五年。这是省委、省政府和市委正确领导的结果，是全市人民团结奋斗的结果。在此，我代表市政府，向全市各族人民，向人大代表、政协委员、各民主党派、各人民团体和社会各界人士，向驻沈解放军、武警部队官兵和中省直单位，向关心支持沈阳建设发展的港澳台同胞、海外侨胞和国际友人，表示诚挚的感谢和崇高的敬意！

　　各位代表，去年我市地区生产总值、一般公共预算收入、固定资产投资等经济指标增速低于全年预期目标。这既有全球经济低迷、国内“三期叠加”的周期性原因；也有我们自身产业结构不合理、自主创新能力不强的结构性原因；还有我们积极落实国家减税降费要求的政策性原因；更有我们认真践行“三严三实”，注重质量、做实数据的主动性原因。

　　同时，我们也清醒地看到，发展过程中还存在着一些体制性、机制性矛盾和问题。一是国有企业缺乏活力，民营经济不大不强，经济发展外向度不高。二是城乡发展不平衡、县域经济仍是短板，交通不畅、环境污染等热点难点问题仍然存在。三是政府在转变职能、优化服务上还有差距，个别部门及工作人员效率低下、办事拖沓，缺乏激情和担当。对此，我们将高度重视，采取有力措施认真加以解决。

　　二、“十三五”时期目标任务和2016年重点工作

　　各位代表，“十三五”时期是全面建成小康社会的决胜期，是全面创新改革的攻坚期，也是推动老工业基地全面振兴的关键期。我们必须贯彻落实“四个全面”战略布局，牢固树立“五大理念”，认真落实中央全面振兴东北地区等老工业基地的若干意见，按照“四个着力”总体要求，贯彻市委十二届十二次全会部署，紧紧围绕“十三五”规划纲要确定的总目标、总任务，大力实施创新驱动发展战略，全面深化体制机制改革，加快产业转型升级，全面提升对外开放水平，深入推进新型城镇化，加强城市建设与管理，推进沈阳经济区一体化，加强生态环境建设，全面提升文化软实力，着力保障改善民生，加快建设国家中心城市，实现沈阳老工业基地全面振兴，确保如期全面建成小康社会。

　　各位代表，今年是“十三五”开局之年。当前，沈阳正处于爬坡过坎的关键阶段，面临着新形势、新挑战。我们要看到，我国经济受多种因素影响，面临的形势更加严峻。我们还要看到，我国经济长期向好的基本面没有改变，国家宏观经济政策效应正在逐步释放。我们更要看到，国家深入实施新一轮振兴东北的重要战略、沈阳成为国家全面创新改革试验区的重大机遇，特别是城市多年来发展积累奠定的雄厚基础、我们拥有一支善谋实干、甘于奉献的干部队伍，还有全市人民期盼和追求发展凝聚起来的强大合力，都是我们推进振兴发展的特殊优势和有利条件。我们一定要抢抓机遇、坚定信心，树立强烈的发展意识和责任意识，以全面创新改革为动力，以提升质量效益为重点，以保障改善民生为主线，以智慧城市建设为载体，统筹抓好稳增长、调结构、促改革、惠民生等各项工作，促进经济社会持续健康发展，确保实现“十三五”良好开局。

　　今年全市经济社会发展的主要预期目标是：地区生产总值增长5.5%，固定资产投资增长6%，一般公共预算收入增长3%，社会消费品零售总额增长8%，城镇居民人均可支配收入增长6.5%，农村居民人均可支配收入增长7%，城镇登记失业率控制在4%以内。

　　(一)坚持创新发展，不断增强发展动力

　　创新是引领发展第一动力。要深入实施创新驱动战略，全面推进创新改革试验，加快建设创新型城市。

　　以科技创新为核心，大力培育发展新动力。加快体制机制创新，以沈阳国家大学科技城为先导，探索建立以企业为主体、“政产学研金”相结合的全链条、贯通式协同创新体系，重点推进沈阳材料与制造国家实验室、沈阳工业技术创新研究院等各类创新平台建设，加快建设东北科技研发创新中心。深化科技体制改革，创新资源配置方式，构建市场驱动的科技研发和成果转化机制，建设区域性科技大市场，打通科技成果资本化、产业化通道。积极开展产业金融创新，支持天使基金、风投基金投资科技企业，鼓励金融租赁、融资租赁发展，大力发展金融市场，努力增强金融服务科技创新和支撑产业升级的能力。实施人才兴市战略，落实盛京人才计划，建立高端人才库，积极引进各类人才和创新团队，构建人才激励保障机制，营造人尽其才、大显其才的良好环境。努力培育“崇尚创新、宽容失败”的文化氛围，让创新成为城市发展的“基因”力量。

　　以创新政府管理体制为重点，努力营造发展良好环境。进一步厘清政府、市场、社会的关系，重新梳理政府职能，科学设置政府机构。组建行政审批局，深化公共资源交易管理体制改革，进一步简政放权、放管结合、优化服务、提高效能。创新绩效考评体系，加大创新创造、绿色发展、文明城市等指标权重，实行智慧化、差异化考核。制定公布涉企收费清单，落实国家正税清费政策，帮助企业降低非生产经营成本，减轻企业负担，为企业搬迁和要素流动创造良好条件。深化商事制度改革，推动大众创业、万众创新，加快推进众创、众包、众扶、众筹等新模式、新业态，让一切创造财富的源泉涌流、活力迸发。

　　以市场化为方向，探索实践国企改革新模式。建立国有资产管理新体制，组建国有资本投资运营公司，开展履行出资人权利试点，放开竞争性国企领导人员管理权和企业投资权、融资权、资产处置权，整合政府各类资产资源，提高国有资本收益水平。分类推进国有企业混合所有制改革，积极促进国有资产资本化、证券化，重点推进盛京银行、沈鼓集团A股上市。建立健全市场化经营机制，开展规范董事会建设试点。积极解决厂办大集体和“壳”企业等历史遗留问题。

　　(二)坚持转型发展，建设制造强市

　　制造业是沈阳的立市之本、发展之基。要以智能制造为主攻方向，促进传统产业升级，培育壮大新兴产业，加快建设具有国际竞争力的先进装备创新制造基地。

　　构建新型工业体系。紧紧围绕“中国制造2025”确定的战略任务和重点领域，打造智能制造装备和航空装备等新增长极，加快提升新一代信息技术、电力装备、汽车、机械装备等优势产业，培育壮大现代建筑产业及专用设备、先进轨道交通装备、新材料、生物制药等新兴产业。创新制造业服务体系，加快建设重点产业技术创新联盟，打造公共研发、工业设计、检验检测等公共服务平台。促进军民优势资源双向流动，建设军民融合示范区。

　　加快传统制造业转型升级。深入推进中德装备园建设，加强与德国工业4.0高效对接，大力发展高端装备制造业。落实“互联网+”行动计划，按照“精准扶持、分类施策”原则，推进80户企业智能化升级。充分发挥产业联盟作用，强化装备制造业上下游配套合作，鼓励骨干企业加快向产品成套化发展、向产业链高端延伸、向服务化转型。促进汽车产业优化升级，推进铁西宝马研发中心、大东北盛技术改造、浑南米拉汽车研发与检测中心、华龙新能源汽车和辽中金杯卡车扩产等项目建设，提升整车配套能力，积极发展新能源汽车。淘汰化解落后过剩产能，让更多要素资源向优势企业、优势产业集聚。开展质量提升、品牌培育、标准化建设行动，提高企业核心竞争力。

　　推动新兴产业发展壮大。做大机器人及智能制造产业，建设沈阳机器人和智能制造创新研究院，突破核心技术和关键零部件瓶颈，进一步拓展应用市场。以机床集团为依托，大力发展高端数控机床。抢抓国家发展干支线飞机和开展重大专项机遇，统筹浑南、沈北、法库航空产业园区建设，重点推进整机装配、结构件制造和通用飞机研发与制造，促进航空产业加速发展。以利源轨道交通、铁路信号等企业为龙头，延长产业链条，做大做强轨道交通装备业。完善现代建筑设计、生产、施工等环节标准化体系，提升现代建筑产业发展水平。推动智能软硬件、传感技术、云计算与大数据、物联网等智慧产业发展，加快发展新材料、生物制药、新能源、节能环保等产业。

　　(三)坚持协调发展，进一步优化经济结构

　　协调是持续健康发展的内在要求。要切实把调整比例、补齐短板、优化结构作为重大任务来抓，在协调发展中拓展空间、增强后劲。

　　优化产业结构。围绕供给侧结构性改革，采取积极措施，提升全要素生产率。以需求升级促进产业升级，做大服务业、做优农业，提高有效供给能力。推动生产性服务业向专业化和价值链高端延伸，促进生活性服务业向精细和高品质转换，加快东北区域金融中心、物流中心、会展中心、商贸中心建设。大力发展金融、物流、会展、电子商务，积极发展总部经济，办好制博会、中国城市规划年会等知名展会活动。以扩大服务消费为重点，改造提升特色街区等传统服务业，大力发展文化、体育、旅游、健康等产业，促进消费结构升级。推进卧龙湖国家级生态旅游示范区创建工作，加大仙子湖、珍珠湖旅游开发力度，挖掘整合旅游资源，打造旅游品牌，建设旅游目的地城市。制定化解房地产库存政策，加强综合配套，推进户籍制度改革、农民工市民化，实行购租并举、活跃二手房市场等措施，推动房地产市场健康发展。推进农业现代化，兑现各项强农惠农富农政策，继续抓好“三带”建设，推进沈阜现代农业示范带建设，提高农业综合效益。加快转变农业发展方式，发展智慧农业，推进现代农业科技成果转化和技术推广。稳步推进土地流转，培育新型经营主体，做大农业产业化龙头企业，大力发展农产品加工业。

　　优化需求结构。全面实施项目带动战略，加大项目的策划、包装和储备力度，做好招商落地、开工建设、达产达效等工作。切实抓好260个重大产业项目、75个新兴产业和新兴业态项目建设，加强项目“全过程、精细化、信息化”管理，加快投资进度，提高投资效率。发挥政府投资引导作用，吸引和鼓励企业、社会投资，加快PPP项目建设，形成多元化投资格局。在稳定传统消费需求的同时，引导消费向智能、绿色、健康、安全方向转变，不断培育新的消费增长点。深入实施“外贸出口五年倍增计划”，促进优势产品和服务出口，不断提升出口规模和档次。

　　优化城乡结构。推进城乡基础设施、社会保障、公共服务一体化，加快以人为核心的新型城镇化建设，稳步推进辽中撤县设区，进一步提高城镇化率。提升产业园区基础配套水平，推进种养加一体、一二三产融合，进一步补齐县域经济短板。支持苏家屯区建设国家产城融合示范区，加快新民市国家新型城镇化综合试点市建设。推进供销社综合改革。深化农村产权制度改革，切实维护农民权益，让农民充分享受城乡一体化发展的成果。

　　推动沈阳经济区协同发展。发挥中心城市集聚辐射作用，创新区域合作机制，推进沈抚同城化发展，携手周边城市强化基础设施互联互通、产业合作、环境保护、要素资源共享和合理配置，共同打造区域协同发展的增长极。

　　(四)坚持建管并重，提升城市治理现代化水平

　　城市建设和管理是实现城市现代化的基础。要坚持以人为本，增强规划的科学性、系统性和权威性，加快智慧城市建设，完善城市功能，不断提高城市发展质量和运行效率。

　　提升基础设施建设水平。以完善城乡基础设施建设体系为目标，全面推进智慧城市、生态环境、畅通工程等重点项目建设，城建计划投资302亿元。加快重大基础设施建设，重点实施城市轨道交通、优化快速路网、打通交通节点、增加跨河通道等项目，加快地铁建设，推进动车公交、空轨公交，打造现代化立体交通体系，提升城市运行保障能力。规划建设城市慢行系统，拓展延长自行车道、人行步道和健身绿道。抓好城区道路整修、公园系统完善升级、农村路网建设、农村环境整治等工程建设。加大城市地下管网建设和改造力度，推进北部供水管网建设，完成南塔配水管线加固，推进雨污分流，加快建设“海绵城市”。

　　实施城市智能化、精细化管理。深化城市管理行政执法改革，建立属地管理、条块结合的管理模式，集中开展市政设施、市容秩序、公用设施等10大专项治理行动。建设城市智能管理系统，提高城市综合治理能力和水平。深入推进“公交都市”创建工作，优化公交网线，加大公交车辆更新力度，加快智能停车场建设，强化城市交通智能化管理，提高城市道路整体通行效率。创新公共服务供给方式，构建政府、企业、市民多元治理模式，提高保洁、绿化、垃圾处理和水电暖气保障等各类公共服务共建、共享能力。加强对城市出入口、城乡结合部等薄弱环节的环境综合整治，不断提升城市形象。提高城市规划水平，保护和传承文化特色、历史遗存、建筑风格等城市印记。

　　(五)坚持开放发展，提高经济发展外向度

　　开放是增强发展活力的重要途径。要实施更加积极的开放战略，坚持双向开放、优进优出，努力打造东北开放新高地。

　　切实提高招商引资质量效益。坚持引资与引智、引技相结合，由追求数量和规模转到注重质量和效益上来。不断强化招商活动的系统化、专业化，充分发挥各类招商平台、商会、协会作用，加大对重点企业、行业和区域的招商力度。以中德装备园建设为依托，不断深化与德国在科技、产业、教育等方面的合作。

　　着力提升对外经贸发展水平。紧紧抓住“一带一路”建设等战略机遇，积极拓展“沈满欧”铁路通道、沈阳陆港和空港通道，加快综合保税区和沈阳空港经济区建设。大力实施“走出去”战略，支持骨干企业发挥竞争优势，扩大国际产能合作。加快境外工业园区建设，深化与“一带一路”节点国家经贸合作，推动装备、技术、标准、服务走出去，培育一批沈阳的跨国公司。

　　努力打造良好投资环境。深化外商投资管理体制改革，扩大国际交流合作渠道和范围。优化沈阳口岸72小时过境免签服务，建立与国际接轨的教育、医疗、卫生等配套服务体系。加强产权保护，维护合作利益。进一步放宽市场准入，加大对中小微企业支持力度，激发非公有制经济活力。保持政策和工作的连续性，信守承诺，进一步提高政府公信力。以政务诚信带动社会诚信，为经济社会发展创造良好的信用环境。

　　(六)坚持绿色发展，提升生态环境质量

　　绿色是永续发展的必要条件。要以创造优良人居环境为目标，大力开展生态修复和环境治理，加强生态保护红线管控，推动形成绿色发展方式和生活方式，促进人与自然和谐共生。

　　大力开展蓝天行动。推动能源和产业结构优化升级，建设清洁低碳、安全高效的现代能源体系，支持绿色清洁生产，推进传统制造业绿色化改造。全力开展抗霾攻坚行动，实施燃煤总量控制、机动车尾气治理、扬尘污染管控、重污染企业搬迁和提高秸秆综合利用率等九项重点工程。加强污染源在线监控能力建设，强化预测预警、科学应对。综合运用经济、法律、行政等手段，严格责任追究，以壮士断腕的决心治理大气污染，努力让城乡人民呼吸上清洁的空气。

　　持续开展绿化提升行动。全面落实绿化提升三年行动计划，大力开展道路、水系、公园、铁路沿线、城市出口等重点区域绿化景观提升工程。进一步巩固提升辽河、浑河、蒲河沿线生态景观环境，建设辽河七星湿地生态景观带。加快推进生态林、景观林、经济林建设，下力气建设东部山区和西北部沙区森林，不断提高森林覆盖率和蓄积量，构建更加完善的城市绿色生态系统。

　　实施城乡水环境治理工程。科学规划利用全域水系资源，实施源头治理、水质提升等工程，建设互通互联的城市网状水系。加快城乡污水处理厂建设，启动东部污水处理厂、南部污水处理厂二期和12处乡镇污水处理设施建设。实施柳河改造，开展新开河、南运河、辉山明渠和满堂河截污、清淤、水利设施等工程，综合治理提升环城运河水系，努力为人民群众提供更多的亲水环境。

　　全面推进节能减排。实施“全民节能行动计划”，倡导节俭、绿色、文明消费。加快节能减排技术开发和推广应用，从源头上控制高耗能、高排放产业。全面落实《沈阳市生活垃圾管理条例》，加大城乡垃圾处理力度。加快老虎冲垃圾焚烧发电厂建设，开工建设大辛、张士垃圾焚烧发电厂，老虎冲餐厨垃圾处理厂、大辛渗沥液处理厂建成并投入运行。推进西部500吨污泥厂扩容、祝家150万吨积存污泥无害化处理工作。

　　大力发展循环经济。强化企业能源资源“小循环”利用。推进法库陶瓷产业园、华润沈本生态循环经济产业园、中德装备产业园等园区“中循环”运行。利用生活垃圾焚烧发电，促进中水循环利用，构建产业、生产生活之间全域“大循环”系统。积极推动国家循环经济示范城市建设。

　　(七)坚持共享发展，建设幸福沈阳

　　共享是发展的目的。要牢固树立以人民为中心的工作导向，把人民群众对美好生活的期盼变成我们的目标行动，实施10大工程，办好民生实事，让城市更美好、人民更幸福。

　　实施就业创业工程。把促进就业作为最大的民生，坚持就业优先战略，实施更加积极的就业政策。提高就业服务网络化水平，加强就业和再就业培训，加大对高校毕业生、农民工、特困群体的就业帮扶力度，构筑政府、高校、社会力量“三位一体”的就业创业服务机制。全面推动“万人创业工程”，落实小额担保贷款、支持创业带头人等创业政策，加快创业孵化基地建设，激发民间创业热情，形成以创业带动就业的良好局面。

　　实施社会保障工程。加快构建城乡一体的社会保障体系，努力实现“五险合一”，提高城乡居民最低生活保障标准，统筹城镇职工医保、居民医保和新农合。推进居家、社区、机构、企事业单位“四位一体”社会化养老服务体系建设，鼓励社会力量兴办养老机构，推广医养结合新型养老模式，新建10个居家养老服务中心和30个社区老人日间照料站。加强大病救助和困难家庭临时救助，加大对独生子女伤残或失独家庭扶助力度，大力发展慈善和残疾人事业，确保兜住社会民生底线。

　　实施脱贫攻坚工程。动员各方面力量参与扶贫开发，构建专项扶贫、行业扶贫、社会扶贫三位一体的扶贫格局。坚持因人因地施策，实施“五个一批”工程，深入开展“城乡互联、结对共建”活动，扎实做好建档立卡贫困人口脱贫工作，确保康平县全面脱贫。

　　实施教育现代化工程。继续增加学前教育公办和普惠性民办学位，推动义务教育优质均衡发展，启动普通高中优质达标建设，提升特殊教育办学水平，推动终身教育向社区和农村延伸。按照市场机制，推进职业教育改革发展，深化校企融合，大力培养高素质技术技能型人才。充分发挥驻沈高校推动经济发展作用，鼓励高校向应用型大学转型。以数字校园为重点，推动区县开展智慧教育建设。落实中考制度改革和现代学校制度，进一步增强教育发展活力。

　　实施医疗卫生提升工程。全面开展“国家卫生城市”和“国家健康城市”创建工作。启动市中医院、浑南现代综合医院、传染病院、口腔医院等新建项目，大力培养全科医生，实施基层医疗卫生机构三年达标建设工程。启动建设人口健康信息平台，加快推进智慧医疗和“医联体”建设。积极推进城市公立医院改革试点，引导支持社会资本参与公立医院改制。稳步推进全面二孩生育政策。

　　实施文化精品工程。创作更多具有地域特色、无愧于时代的优秀作品，讲好“沈阳故事”。加快文化体制改革，培育骨干文化企业，打造文化支柱产业。扎实推进“艺术惠民双百万工程”，加大城乡文化基础设施建设投入，加强历史文化遗产保护，加快沈阳妇女会馆、青少年宫、美术馆、职工之家劳模纪念馆、盛京文化智慧城等项目建设，举办“中国·沈阳国际合唱节”，培育城市文化品牌。弘扬正能量，正确引导心理预期，培育荣誉文化，塑造城市精神。

　　实施体育惠民工程。深入实施全民健身战略，加大各类体育设施向社会开放，实施体育活动和器材进社区、进学校、进村屯、进机关、进企业。促进体育休闲消费，加快发展体育产业，建设休闲体育产业集聚区。将“沈阳马拉松”打造成国际金标赛事。开展以足球为重点的丰富多彩的全民健身活动，建设沈阳足球博物馆，推动校园足球发展，办好“哥德杯”等国内外大型赛事，将足球打造成沈阳的靓丽名片。

　　实施和谐社区工程。加快区域性社区服务中心建设，不断健全社区治理、服务、保障三大体系。继续推进“智慧社区”建设，建立人口社会信息系统，逐步建成市、区、街、社区四级服务网，形成小事不出社区、大事不出街道、难事妥善解决的便捷高效服务机制。完善社区事务“一口式”受理和网格化管理平台，提升社区政务服务、公共服务、商业服务功能，提高社区综合管理和惠民服务水平。

　　实施公共安全保障工程。畅通群众利益协调、权益保障渠道，加强社会稳定风险评估，做好信访工作，有效化解社会各类矛盾。提高公共应急处置和社会治安立体防控能力，加强自然灾害监测预警，继续开展危险化学品、食品药品、交通消防、特种设备、人员密集场所的安全专项整治，落实安全责任体系，强化安全监管，有效防范重特大安全事故发生。全力做好国家食品安全城市创建工作。

　　实施人居环境改善工程。加快推进棚户区改造等保障安居工程，完成2.55万户城市棚户区、8290户农村危房改造。推广沈河“楼道革命”经验，全面开展楼道整治行动。实施553个旧住宅区环境改造和1000万平方米节能暖房工程。继续推进城乡饮水安全工程建设。改造2030公里村内道路，新建195公里自然村油路，整修500公里农村公路，完成2.6万座农村改厕任务，创建220个达标村和5个示范样板村，加快建设美丽宜居乡村。

　　(八)坚持效能为先，加强政府自身建设

　　政府效能是衡量和检验政府工作的重要标准。要坚持转变政府职能，积极构建运行规范、决策科学、执法严格、服务高效、监管有力的政府管理新格局。

　　加强法治政府建设。牢固树立法治理念，依法设定、行使、制约、监督权力。推进综合执法改革，进一步规范行政执法自由裁量权，严格、规范、公正、文明执法。自觉接受市人大及其常委会的依法监督、市政协的民主监督和社会各方面监督，认真办理人大代表建议、议案和政协提案。做好普法宣传，提高全社会学法、尊法、守法意识。

　　努力转变工作作风。加强对各级政府领导干部实绩考核，强化责任担当，激发工作热情。建立重点工作督查评估机制，保障各项决策部署都得到有效落实。深化政务公开，打造“透明”政府。推行窗口部门延时服务，切实做到便民利民。深入实施“两转一优”专项行动，进一步提高服务水平和工作效率。

　　大力加强廉政建设。认真学习并严格执行《中国共产党廉洁自律准则》和《中国共产党纪律处分条例》，切实履行政府党组党风廉政建设主体责任，全面落实“一岗双责”。建立健全长效机制，严格执行中央八项规定和省、市相关要求。强化对行政权力的制约与监督，树立廉洁自律政府形象。

　　各位代表，我们又站在了新的历史起点上，新的目标承载着新的梦想，新的征程充满了新的希望。实现沈阳新一轮振兴发展的光荣使命，鼓舞我们开拓创新；全市人民的信任期望，激励我们奋勇向前。让我们更加紧密地团结在以习近平同志为总书记的党中央周围，在市委的正确领导下，在市人大及其常委会依法监督和市政协民主监督下，凝心聚力，只争朝夕，敢为人先，拼搏进取，为加快沈阳老工业基地全面振兴，为全市人民生活更加幸福美好而努力奋斗！
